# Landscape mapping at sub-Antarctic South Georgia provides a protocol for underpinning large-scale marine protected areas

**DOI:** 10.1038/srep33163

**Published:** 2016-10-03

**Authors:** Oliver T. Hogg, Veerle A. I. Huvenne, Huw J. Griffiths, Boris Dorschel, Katrin Linse

**Affiliations:** 1 British Antarctic Survey, Natural Environment Research Council, High Cross, Madingley Road, Cambridge, CB3 OET, U.K; 2 National Oceanography Centre, University of Southampton Waterfront Campus, European Way, Southampton SO14 3ZH, Southampton, U.K; 3 University of Southampton, Waterfront Campus, European Way, Southampton SO14 3ZH, U.K; 4 Alfred Wegener Institute, Helmholtz Centre for Polar and Marine Research, Mitte, Am Alten Hafen 26, 27568 Bremerhaven, Germany

## Abstract

Global biodiversity is in decline, with the marine environment experiencing significant and increasing anthropogenic pressures. In response marine protected areas (MPAs) have increasingly been adopted as the flagship approach to marine conservation, many covering enormous areas. At present, however, the lack of biological sampling makes prioritising which regions of the ocean to protect, especially over large spatial scales, particularly problematic. Here we present an interdisciplinary approach to marine landscape mapping at the sub-Antarctic island of South Georgia as an effective protocol for underpinning large-scale (10^5^–10^6^  km^2^) MPA designations. We have developed a new high-resolution (100 m) digital elevation model (DEM) of the region and integrated this DEM with bathymetry-derived parameters, modelled oceanographic data, and satellite primary productivity data. These interdisciplinary datasets were used to apply an objective statistical approach to hierarchically partition and map the benthic environment into physical habitats types. We assess the potential application of physical habitat classifications as proxies for biological structuring and the application of the landscape mapping for informing on marine spatial planning.

Rates of species extinction currently exceed those of speciation, leading to a decline in global biodiversity^1^,^2^,^3^. Anthropogenic activities are significant drivers of this decline^4^, with the marine environment experiencing significant and increasing pressure from extractive industries such as fishing, marine deposit mining, and oil and gas extraction, activities, which are increasingly proliferating into deep-sea environments^5^. Furthermore, the world’s oceans are becoming warmer and more acidic^6^, driving changes in the distributions of marine species^7^, and impinging on ecosystem services^8^,^9^.

Under financial, political and social constraints, two key questions that precede any conservation strategy are how and where to prioritise limited resources and effort to maximise the scientific robustness of conservation strategies in an increasingly exploited, yet still poorly understood marine environment. In response to international frameworks such as the Convention on Biological Diversity^10^, conservation efforts have increasingly prioritised the protection of regions and ecosystems as a holistic and geopolitically achievable approach to conservation. In recent years this has led to the establishment of several large-scale (10^5^–10^6^  km^2^) marine protected areas (MPAs) at sites including Ascension Island (2016), Pitcairn Island (2015), South Georgia and South Sandwich Islands (2012) and the Chagos Archipelago (2010). The basis of many of these MPAs centre on the presence of particularly vulnerable, keystone, or charismatic species; the presence of large numbers (or proportions) of endemic species; and the presence of high biodiversity across taxonomic levels (i.e. biodiversity hotspots)^11^,^12^. The scientific underpinning for the creation of such vast reserves is based predominantly on *in situ* data such as biological sampling (e.g. by trawling, dredging and diving) and direct observation (e.g. by divers or camera systems). *In situ* sampling and observation, however, is intrinsically expensive, time-consuming, and logistically difficult, especially when attempting to cover large areas, inaccessible or remote regions. As such, biogeographical knowledge of the marine environment through *in situ* sampling is typically spatially very patchy and in some instances, notably the deep sea, virtually non-existent.

This paucity in knowledge is particularly problematic for the scientific underpinning of large-scale MPA designations. Frequently, ambitious political and social demand for high-impact, low-cost and large-scale management solutions conflict with scientific data that only support designation on localised scale, or in a generalised way (i.e. a region is known to have high faunal diversity but little is known about its distribution). As a consequence, MPA designation tends to be scientifically underpinned by the data that are available, but not scientifically driven in their design. By default the spatial configuration of many MPAs therefore tends to be delineated by geopolitical boundaries (e.g. extent of Exclusive Economic Zones) and located in often remote overseas territories that elicit minimal stakeholder opposition.

In response to the challenge of limited biological data, habitat mapping can be used to greatly increase the value of limited *in situ* data for marine conservation, by inferring associations between ground-truthed sample data, remotely sensed geophysical data (e.g. topography; surficial geology), and physical environmental data (e.g. oceanography). In this way, in the absence of sufficient biological data, geophysical and biophysical attributes such as depth, substrate type, and geomorphology can act as useful surrogates for biological communities or assemblages^13^. Analysis of the interaction between physical and biological variables helps create a more comprehensive spatial and temporal understanding of how biodiversity is structured across a region, and can help constrain the bio-physical processes driving this structuring. Furthermore remotely sensed data are typically orders of magnitude faster and cheaper to collect per unit area than ground-truthed sample data^14^.

Marine landscape mapping is a form of *top-down* habitat mapping that delineates and describes the marine environment on the basis of physical characteristics. Biological data are then used to ground-truth the map to test how effective proxy habitat classification is for modelling biological distributions. Marine landscape mapping is often adopted as a broad-scale approach to habitat classification^15^,^16^, but is a technique with application across spatial scales^17^,^18^,^19^. Through this approach, different geomorphic features such as canyons, pinnacles, rocky ridges and muddy basins can be mapped, either for their own intrinsic conservation value^20^, or for use as proxies of particular types of benthic habitats^21^. This makes it a technique with potential application for assessing risk to benthic communities and in the proposing of MPAs designations.

The waters surrounding the sub-Antarctic island of South Georgia are one of the largest recently designated MPAs, and include one of the largest and most isolated continental shelf areas in the Southern Ocean. A combination of early separation from a continental land mass, a large shelf area, a high degree of geographic isolation, and the proximity of nutrient-rich currents have led to the evolution of a biologically rich and distinct island^22^. In 2012 the region (including the South Sandwich Islands archipelago to the south-east) was designated an IUCN category IV marine protected area, covering a total area of over one million km^2,^^23^.

The regional waters around South Georgia represent a particularly good model system in which to develop, test, and implement a landscape mapping protocol. The region is one of a number of recent additions to the list of extremely large-scale MPAs. There has been no concerted effort (expeditions to the region) with the specific purpose of mapping physical habitats or biotopes. As such, all data from the region are (from a habitat mapping perspective) based on opportunistic historical data collection. This is important in developing a protocol that has transferability to other marine systems, as the suite of data available at South Georgia is comparable with most other potential large-scale MPA sites. The existence of a number of comprehensive datasets including seabed geophysical data; modelled oceanographic data, sediment data, and an extensive biological dataset provides an ideal opportunity for interdisciplinary habitat mapping. In addition to being a good location to test landscape mapping models, it is also an internationally important site to understand and conserve because of its speciose and unique benthic fauna, commercially important fisheries and the presence of a globally important population of higher predators^12^,^22^.

The aim of this study is to develop a fully automated and objective marine landscape mapping protocol that adopts a hierarchically nested clustering approach to classify physical habitat types across spatial scales. This protocol should be robust, repeatable and have universal application for other proposed or established marine protected areas. It should also enable assessment of the ecological relevance of physical habitat classifications, their application as proxies for benthic biological structuring, and as such the application of the landscape mapping protocol for informing marine spatial planning.

## Methodology

### Study area

**Figure 1:**
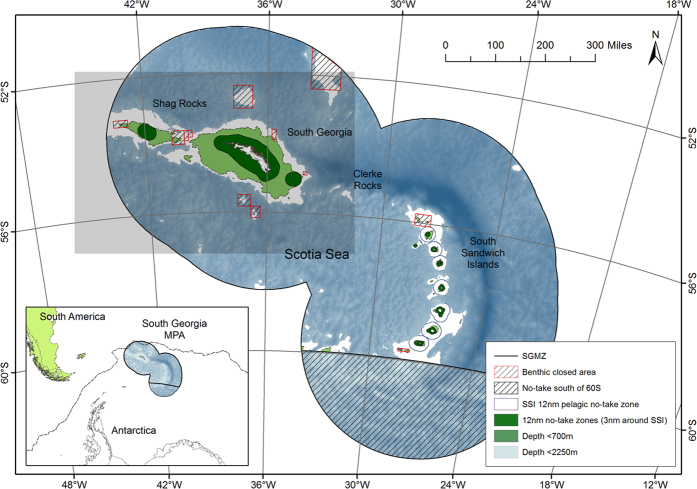
The South Georgia and South Sandwich Island marine protected area. Dark green areas demark the no-take zones around South Georgia, South Sandwich Islands, Shag Rocks and Clerke Rocks. Light green indicates depths less than 700 m in which bottom fishing is prohibited. Hashed boxes with red border are additional benthic closed areas established in 2013 in which bottom fishing is prohibited. The purple borders around SSI are a 12nm pelagic no-take zone. The large black hashed area south of 60° S falls within the SGSSI Maritime Zone in a region for which no fishing licenses are issued. In all other regions of the SGMZ bottom fishing is prohibited with the sole exception of the narrow pale blue region which includes the depths between 700m and 2250m. Within this region bottom fishing is permitted by license. The region of interest for this study is delineated by the grey shaded box. The inset shows the position of the South Georgia and South Sandwich Islands MPA relative to South America, the Antarctic continent and the Polar Front (dashed line). Figure was created using ArcGIS (version 10.1 [www.esri.com/software/Arcgis]). Background bathymetry is The GEBCO_2014 Grid, version 20141103 (http://www.gebco.net).

The greater South Georgia region, which forms the focus of this paper, is defined here by a bounding box of ~900 km (45°E to 19°E) by ~580 km (63°S to 50°S), covering an area of 530,000 km^2^ (Fig. 1). The region includes the South Georgia shelf, the Shag Rock shelf (to the west of South Georgia), the surrounding continental slopes and adjacent deep sea. It does not, however, extend to include the South Sandwich Islands region of the SGSSI MPA. The rationale for this geographical delimitation at the expense of covering the entire > 1 million km^2^ of the SGSSI MPA was based on the limited spatial extent of input datasets (notably the modelled oceanographic dataset).

### Data compilation

#### Bathymetry gridding process

We constructed a new high resolution (100 m) bathymetric grid of the South Georgia continental shelf, slope and surrounding open-ocean. The grid was derived from multibeam and single-beam echo-sounding data compiled from numerous research institutes, universities and commercial fisheries (see Supplementary materials Table 1). Given the diverse range of data sources, the echo-sounding data varied greatly in terms of collection method, data quality, and file format. As such the process of incorporating different data streams into the compilation varied depending on their provenance.

**Figure 2:**
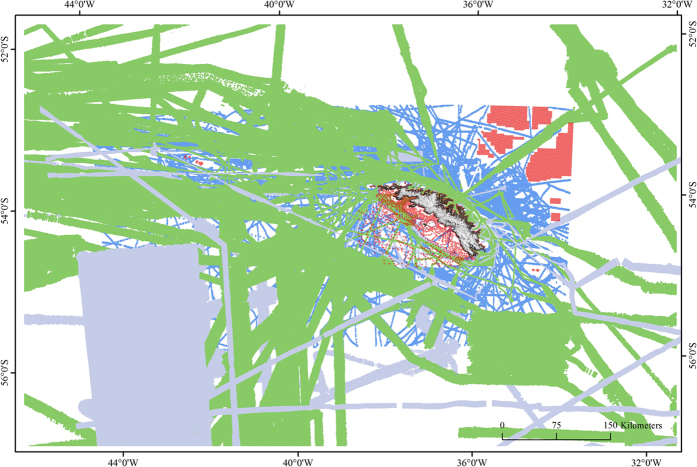
The spatial coverage of data sources used in the bathymetry compilation. Multi-beam data derived from BAS cruises are shown in green; AWI and other multi-beam (see Supplementary materials Table 1 for sources) are shown in grey; single-beam data is shown in blue and UK Hydrographic Office and coastline data is shown in red. For the remaining white areas The GEBCO_2014 Grid, version 20141103 (http://www.gebco.netdata) was used. Figure was created using ArcGIS (version 10.1 [www.esri.com/software/Arcgis]).

Data curated at the BAS Polar Data Centre and collected during 36 research cruises between 1994 and 2013 were gridded at a 100m resolution using MB-System^23^. Unprocessed data from recent BAS cruises to the region were cleaned using CARIS HIBS & SIPS (version 8.1) to remove erroneous points. Data collected by the Alfred Wegener Institute, Bremerhaven (AWI) over the course of 22 cruises to the region between 1985 and 2013 as well as data collected on a recent R/V Nathaniel B. Palmer cruise were processed and gridded to 100m resolution using Fledermaus (version 7.0). Single-beam bathymetry data were obtained from the UK Hydrographic Office (UKHO), BAS and commercial fisheries vessels. The spatial coverage of respective datasets is summarised in Fig. 2.

As with a number of previous bathymetric compilations^24^,^25^,^26^ we used TOPOGRID to grid the digital elevation model. TOPOGRID is an interpolation function in ArcGIS (version 10.1) built around the ANUDEM algorithm^27^,^28^, which has been shown as an effective approach to integrating spatially discontinuous data with different sampling densities whilst minimising standard error in the model^26^. The ANUDEM algorithm runs over multiple iterative cycles. It starts by gridding data at a coarse spatial resolution before interpolating at successively finer resolutions, until the pre-defined resolution (in this case 100m) is reached. TOPOGRID is particularly well-suited to constraining fine-scale topographic features whilst imposing constraints to prevent erroneous sinks being formed in the output digital elevation model (DEM)^29^.

The quality of the data varied between surveys as a result of a number of factors including the echo-sounder equipment used, the calibration of this equipment (i.e. the use of different sound-velocity profiles), the sea state conditions at the time of data collection, and the degree and quality of data post-processing and cleaning. This variability was manifest in two key regards: the effective spatial resolution of the data (i.e. at what spatial scale the bathymetry could be rendered at), and the presence of acquisition artefacts (artificial ‘sinks’ or ‘peaks’) in the data.

TOPOGRID reads data as point ‘xyz’ (longitude, latitude, depth) records for each data source. Creating a bathymetric compilation from a point cloud is inherently problematic given the variability in the quality of the data from different sources. Specifically, this is a problem when regions of data points from different sources (which may vary in spatial resolution by 10 s or 100 s of meters) overlap. The algorithm will treat each point as equally valid, and draw a spline between them, potentially creating steps in the data as it moves between points from different sources. To remove this artefact, we ranked our datasets hierarchically based on data quality. Using spatial coverage masks of each dataset, regions of overlapping data were cut from all masks except for the mask representing the best-quality data available from that particular region. In this way we created a continuous mosaic of masks for the entire South Georgia region with no overlapping parts and only the best available data representing each region. To avoid artificial ‘steps’ in the data, a 500m buffer was used to create a region of no-data on the boundaries between different data layers. This had the effect of ‘smoothing out’ any abrupt changes at the boundaries of adjoining data layers during interpolation. These masks were then used to extract the respective point data to input into TOPOGRID.

Ideally, only cleaned data would be input into the interpolation. Given the number of data points involved in an analysis of this scale ( > 1.0 × 10^8^), however, this was impractical. After gridding the data with TOPOGRID, the new interpolated 100m resolution raster data was therefore overlaid with the point cloud of original input data. Points that were deemed to be creating false peaks and sinks were manually removed using the ArcGIS Editor toolbox. After this iterative process, the final DEM was considered to be as accurate as possible, with a minimum amount of sampling artefact but without excessive data smoothing.

#### Bathymetry Derivatives

The new 100 m resolution DEM was used to calculate five bathymetry derivative datasets, which alongside oceanographic and net primary productivity data (summarised in Table 1) formed the basis for the landscape mapping analysis. These bathymetry derivatives included measures of slope angle, seabed rugosity, topography, aspect and curvature.

Slope was calculated using LandSerf (version 2.3) multi-scale analysis. To ‘smooth out’ sampling artefacts and noise in the data, the effective resolution of the slope was reduced by introducing a window scale of 10 grid cells (i.e. 1000 m) with an inverse linear distance decay, whereby the analysis takes into account the slope value of surrounding cells (in this case with a diameter of 10) to give greater importance to cells closer to the target cell. The effect of this is to remove finer-scale variation in slope morphology but retain larger topographic features.

Topographic position index (TPI) was calculated using Land Facet Corridor Tools extension for ArcGIS. TPI provides a measure of whether a cell is positioned on a peak, in a depression, or in a region of constant gradient (flat or constant slope) relative to the surrounding cells. It can account for local scale topography versus broader-scale features by changing the size of the window of reference. For this analysis a window size of 10 was used.

Terrain ruggedness index (TRI) was calculated using SAGA GIS Terrain Analysis Morphometry tools as a measure of rugosity. TRI is calculated as the square root of the sum of squared difference between the bathymetric value of a cell and its 8 surrounding cells.

Aspect was calculated using the spatial analyst toolbox in ArcGIS. It provides a measure of the geographical orientation of a region as a circular data variable, from which we created two variables: northness = cos (aspect*π/180), as a measure of orientation on the north-south axis, and eastness = sin (aspect*π/180), as a measure of orientation on the east-west axis.

Profile curvature was calculated using the spatial analyst toolbox in ArcGIS. It is a second derivative index of bathymetry that measures the surface shape of the seabed in the steepest downhill direction quantifying the rate at which slope gradient changes. Regions with constant gradient return a value at or approaching zero, concave and convex slopes return large negative and positive values respectively.

#### Other abiotic variables

Oceanographic data were derived from a high-resolution 3D hydrodynamic model of the South Georgia shelf and adjacent open-ocean^29^. The model is based on the Proudman Oceanographic Laboratory Coastal Ocean Modelling System (POLCOMS) providing salinity, temperature, current magnitude and current direction data at a spatial resolution of 2.8km. Surface and seabed data, taken as a summer (Dec–Feb) and winter (Jun–Aug) mean were averaged over three years (1999–2001). The model is validated against an extensive CTD dataset at South Georgia, shore-based tide gauges, and satellite temperature data. It has been shown to be particularly good at resolving tidal processes, topographically steered currents, and freshwater fluxes from island runoff^29^.

Satellite derived net primary productivity data (NPP) were accessed through Oregon State University (http://www.science.oregonstate.edu/ocean.productivity/). Here NPP is defined as a function of chlorophyll, available light, and photosynthetic efficiency. The data are derived from the Vertically Generalized Production Model (VGPM)^30^, MODIS surface chlorophyll concentrations (Chl_sat_), MODIS 4-micron sea surface temperature data (SST4), and MODIS cloud-corrected incident daily photosynthetically active radiation (PAR). Euphotic depths are calculated from Chl_sat_. The data were extracted as monthly means over a five year period (2010–2014) with a grid cell resolution of 1/12 degree of latitude (~9275 m) by longitude (~5465 m). R (version 3.1.2) was used to define the geographic region of interest, create a data matrix of each month’s mean NPP, and transpose this into a raster dataset with the correct geographic projection and each grid cell pixel representing a mean NPP for five-year of monthly data.

To standardise the spatial extent and resolution of each input variable, each dataset was converted to the same file format (.img), spatial units (meters) and projection (South Georgia Lambert Conic Conformal, WGS1984), with each raster resampled using nearest neighbour analysis to the same spatial resolution. For the oceanographic and satellite primary productivity data which had coarser spatial resolutions (see Table 2), data were resampled to 100m using ArcGIS spatial analyst spline (with barrier) interpolation. Spline with barrier interpolation was selected on the basis of its suitability for environmental variables that change over gradients. The spline barrier used was a polygon of the coastline of South Georgia to prevent values being interpolated across the physical boundary of the island.

#### Landscape Mapping

The statistical approach to mapping marine landscapes presented in this study is based on an unsupervised mapping protocol developed for shallow water shelf environments^18^ and subsequently adapted for use in the high–resolution analysis of submarine canyon systems^19^.

The protocol for landscape mapping can be summarised in five steps. (1) principal component analysis (PCA) of the gridded environmental variables; (2) determination of the optimal cluster solution; (3) *K*-means clustering of the principal components; (4) plotting the optimal cluster solution as a landscape map and assigning environmental meaning to each cluster based on the relationship between the original environmental variables and each cluster; (5) assessment of the stability of the clustering solution based on calculation of membership values and corresponding confusion indices^31^.

The software used for PCA, *K*-means clustering, cluster validation, and raster map creation was R version 3.0.0. Given the large size of the South Georgia datasets, this was run remotely on the University of Southampton *Iridis4* high performance cluster computing system high memory nodes.

#### Principal component analysis (PCA)

Principal component analysis was conducted on 19 abiotic environmental variables (Table 1). The input variables were all standardised to have zero-means and unit variance, thus giving them equal weight in the PCA. PCA reduces the input variables down to a new set of linearly independent variables called principal components (PCs) that account for most of the variance observed in the original data^32^. It acts as an objective means of data reduction when dealing with multiple input variables without the need for an *a priori* assessment of which variables should form the basis of the analysis^33^, whilst removing collinearity in the data. We followed the Kaiser-Guttman criterion^32^ retaining only PCs with Eigen values greater than one. In order to maximise the independence of the PCs and simplify the interpretation of the factor loading pattern, a varimax rotation of the PCs was computed. These rotated PCs were the input for the *K*-means analysis.

#### *K*-means clustering and defining optimal number of clusters

The *K*-means clustering algorithm is a widely used method for partitioning marine environmental data^18^,^19^,^34^,^35^*. K*-means works by partitioning *n* observations into a pre-defined *k* number of clusters whereby each observation is assigned to the cluster that minimises the distance of that point to the cluster centroid. The most subjective element in *K*-means clustering is the requirement for the input of a predefined number of clusters into the algorithm. There are several indices that can be used to define the optimal number of clusters^36^, with many available in *R* through the *NbClust* package^37^. We used two separate indices that have been shown to be offer effective solutions. The C-H criterion^38^ evaluates the validity of different cluster solutions based on the proportion of the total variance explained by variance between clusters (between group sum of squares - SSB)^36^. The second approach was an *elbow* method which plots variance within clusters (within group sum of squares – SSW) against an increasing number of *K*-means clusters (here ranging from 2 to 15). As the number of clusters increases the SSW decreases. The optimal cluster solution is defined by a change in gradient or elbow in the graph representing the point at which increasing the number of clusters further will not greatly reduce SSW.

#### Marine landscape and confidence map

Once the optimal cluster solution was decided, the results of the subsequent *K*-means analysis (predefined for number of clusters) were converted to a raster grid in R and exported to ArcGIS to create a marine landscape map of the region at the same spatial resolution and projection as the original abiotic input variables. Boxplots of the abiotic input variables against the *K*-means cluster solutions were used to characterise and offer interpretation of the environmental conditions driving the classification of each cluster. Interpretation of these boxplots was used to assess the performance of the automated landscape classification in terms of the perceived ecological meaningfulness of the classifications. Given that all input variables were standardised at the start to provide them equal weight in the PCA analysis, qualitative assessment was made as to whether any of the variables were disproportionately influencing cluster classification and if so whether these needed to be removed and the analysis re-run.

Once the final clustering solution was achieved, the stability of the clusters was assessed by creating a separate set of cluster membership maps. Cluster membership is a methodology adapted from fuzzy *K*-means classification^19^,^31^,^39^, and is defined by calculating the relative inverse distance squared in attribute space between each individual data point and the centroids of all the *K*-means clusters. The sum membership value to all centroids of any given point equals 1. As such, a data point with a high membership value (approaching 1) would indicate that point is dominated by membership to one cluster. A data point with lower membership values, spread over a number of clusters, is less well characterised by membership to its *K*-means designated cluster. Membership value is expressed by formula 1:


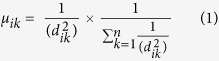


where *μ*_*ik*_ is the membership value of the i^th^ data point to cluster *k*, *n* is the number of clusters, and *d*_*ik*_ is the distance between data point *i* and cluster centroid *k*. Using the highest and second highest membership value for each data point, the confusion index can be calculated for all points to offer a quantitative measure of the uncertainty associated with the classification of each point into clusters by *K*-means. The confusion index is calculated by formula 2:


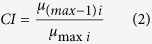


where *CI* is the confusion index, *μ*_max *i*_ is the membership value of the cluster with maximum *μ*_*ik*_ at locat*i*on *i*, and *μ*_(max−1)*i*_ is the second largest membership value at the same *i* location. The CI of any given point can range between 0 and 1, with uncertainty in the *K*-means clustering solution between two or more clusters at that location increasing as CI approaches 1.

#### Nested-hierarchical clustering

Once a stable landscape map is produced which minimises within-cluster variance, and is qualitatively assessed to offer a good representation of the physical marine landscapes of the region, the landscape mapping protocol can begin again. This time the analysis is delineated by, and done in isolation for each of the cluster solutions derived from this first iteration. In this way a nested hierarchical clustering structure for the region is created, whereby each successive level of the hierarchy provides progressively more detail on the physical environment characterising each region. This method of hierarchically nested clustering provides a physical habitat classification system that is specific to a given region of interest. This is in marked contrast to more generalised models of habitat classification^40^, which offer a one-size-fits-all solution that is not always ecological meaningful across all marine environments (e.g. deep-sea environments^41^). This hierarchical approach also offers the opportunity to assess environmental conditions across spatial scales and in turn the interaction between faunal communities and environment over these different scales.

## Results

### Bathymetric Compilation

**Figure 3:**
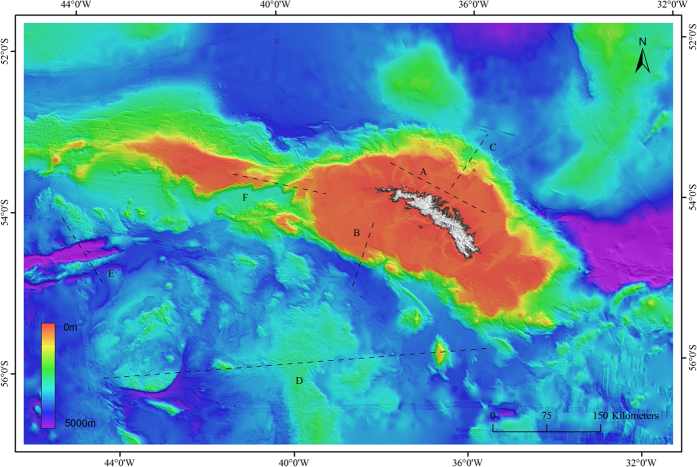
New bathymetric compilation for South Georgia gridded to a spatial resolution of 100 m. Transects (**A–F**) denote depth-profile plots shown in Supplementary materials Fig. 1. Figure was created using ArcGIS (version 10.1 [www.esri.com/software/Arcgis]) TOPOGRID (Spatial Analyst Tools) to grid datasets listed in Supplementary materials Table 1.

The new 100 m resolution South Georgia Bathymetric Dataset (SGBD) (Fig. 3) covers an area of 530,000 km^2^, of which 54.4% (288,470 km^2^) is derived from multibeam data, 10.2% (54,022 km^2^) from singlebeam data and the remaining 35.4% (187,508 km^2^) is derived from The GEBCO_2014 Grid, version 20141103 (http://www.gebco.netdata). The SGBD offers significant improvements on the pre-existing bathymetric knowledge of the region^24^, specifically in terms of the inclusion of new high resolution datasets in the interpolation, the resulting increased spatial resolution of the grid (100m), and its wider geographical extent. The grid is projected in Lambert Conformal Conic (LCC) projection optimised for South Georgia to give a more realistic representation of the shape of topographical features.

The high resolution of the SGBD enables us to better constrain fine-scale topographic features. The shelf is dominated by deeply incised glacial cross-shelf troughs (Fig. 3), extending from coastal fjords, in most cases to terminal moraine fields at or near the shelf break. The troughs form deep (100–160 m) and narrow depressions (~2 km) that converge further offshore to form large (up to 20 km) canyons cut into the shelf. The higher resolution grid enables us to delineate the extent of the shelf itself far more accurately, and pick out in much sharper detail the shelf break, including the presence of complex gully systems on the steep continental slope. The SGBD covers a bathymetric range of 0–5500 m. The inclusion of the deep sea in the compilation reveals that these regions are not homogenous but exhibit complex and sudden changes in topography (Supplementary materials Fig. 1). This includes geological features with some of the steepest slopes in the region (drop-offs from 2500 m to > 4500 m with slope angle > 45°).

### Landscape Mapping

#### PCA

Principal component analysis was conducted using 19 abiotic input variables (Table 1). After running the entire landscape mapping protocol and conducting a qualitative assessment of the output map however, aspect was excluded from the analysis as it was creating ecologically meaningless clusters in the deep sea (i.e. regions > 3000m depth with very low slope gradients but different orientation). As such the principal component analysis was re-run with the remaining 17 abiotic variables from Table 1. Retaining only PCs with Eigen values greater than 1, PCA resulted in six PCs, explaining 81% of the total variance. The rotated components matrix (Table 2) shows the factor loads which explain the correlation between Varimax rotated PCs (with Eigen values > 1) and the original abiotic input variables and excludes any factor loads < 0.3. Assessment of the rotated components matrix showed only one variable (depth) had a high factor load for multiple PCs, all other variables had an exclusive relationship with specific PCs. PC 1 had high loads (r < −0.6 or r > 0.6) for the variables depth, seabed temperature range and seabed salinity; PC 2 for current magnitude (specifically on an easterly axis); PC 3 for current on a northerly axis; PC 4 for slope gradient and terrain ruggedness; PC 5 for depth and seabed temperature; and PC 6 for curvature and topographic positioning. Primary productivity was not related to a specific PC, but did play a role in the distinction between classes (see below).

#### *K*-Means

**Figure 4:**
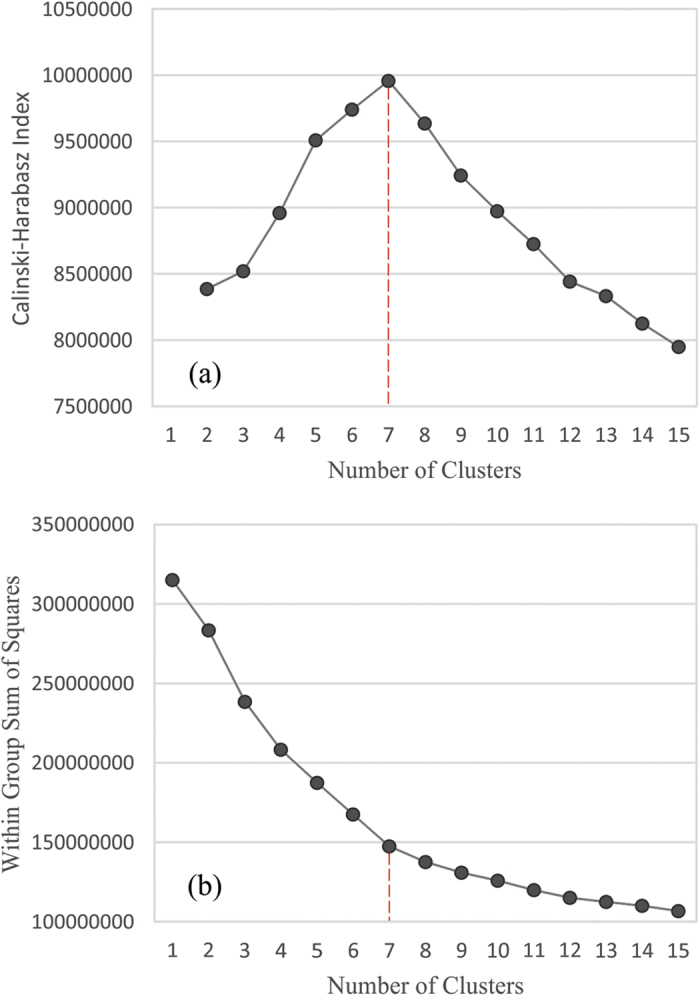
Optimal *K*-means cluster solution. Calculated as, (**a**) the number of clusters versus the Calinski-Harabasz (C–H) criterion, whereby the optimal cluster solution corresponds to the first local maximum of the C-H value; and (**b**) the number of clusters versus the within group sum of squares based on Varimax rotated PCs, whereby the optimal cluster solution is identified by an ‘elbow’ or change in the gradient of the slope. For both indices the best solution is identified as 7 clusters (marked in red). Figures created using R (version 3.0 [www.r-project.org]).

A total of 52,996,140 grid cells with six rotated PC variables were clustered using *K*-means, in a cascade from two to fifteen cluster solutions. The resulting within group sum of squares were plotted against number of clusters (Fig. 4a). Using the ‘elbow’ method, where a change in the gradient of the graph identifies the optimal cluster solution, seven clusters were assessed to be a good fit for the data. To validate this result, the C-H criterion was applied to the data with the same cascade in cluster solutions (Fig. 4b). A clear optimum of seven clusters was found, hence a final *K*-means clustering using seven clusters was carried out.

#### Marine landscape

**Figure 5:**
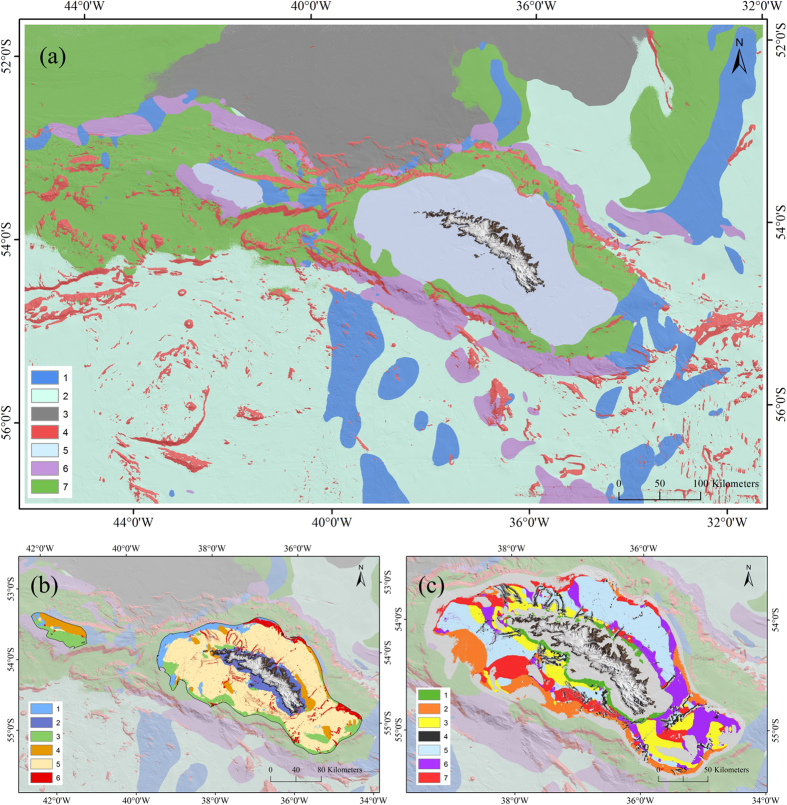
Hierarchically nested marine landscape maps. Showing (**a**) distribution of 7 cluster classes across the whole study region as defined by k-means cluster analysis; (**b**) re-clustering of cluster 5 taken from first k-mean partition (Fig. 5a) whereby the shelf (previously a single cluster) is now split into 6 sub-clusters; and (**c**) re-clustering of cluster 5 - sub-cluster 5 (Fig. 5b) whereby sub-cluster 5 is partitioned into 7 further third-tier clusters. Data for figures gridded in R (version 3.0) and visualised using ArcGIS (version 10.1 [www.esri.com/software/Arcgis]).

**Figure 6:**
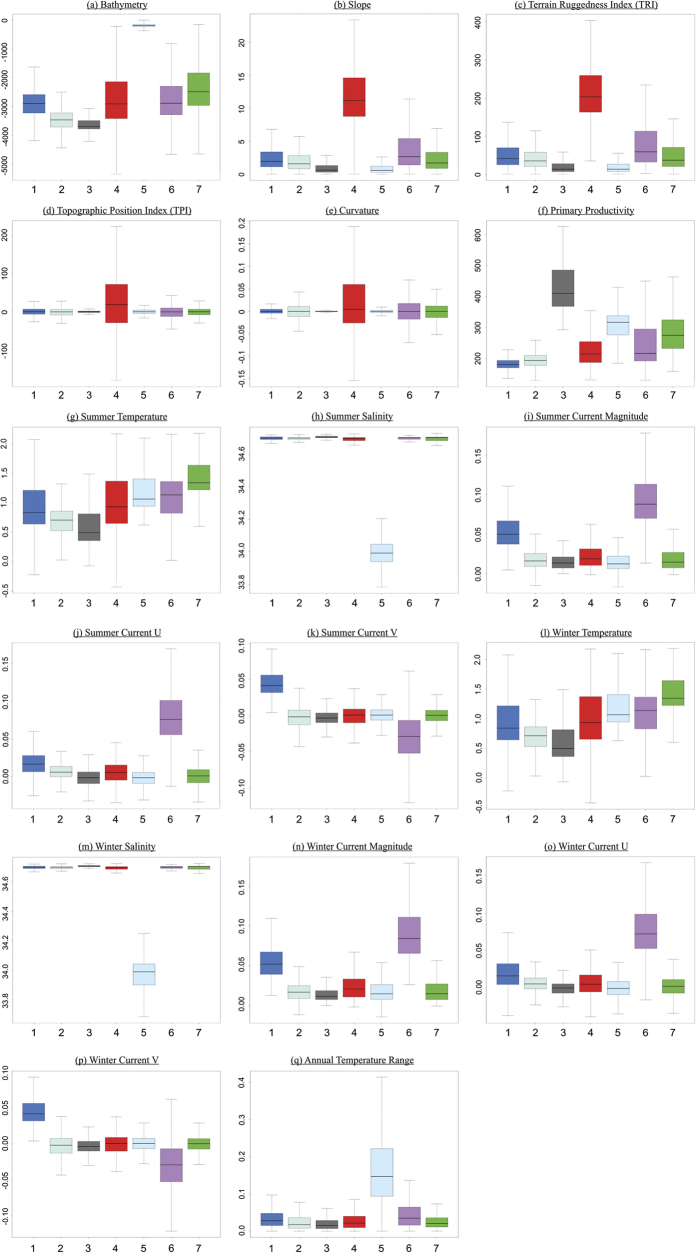
Box plots of *K*-means derived clusters versus 17 original abiotic variables. The x-axis denote the seven k-means clusters, and the y-axis the respective units of each original abiotic variable. Descriptions of each variable including their units are summarised in Table 1. In each box plot the middle line represents the median, the upper and lower extent of the box represent the first and third quartiles. The whiskers are the maximum and minimum observed values (excluding statistical outliers - values > 1.5 x the interquartile range). Box plot colours denote the corresponding landscape map cluster colours from Fig. 5a. Figures created in R (version 3.0 [www.esri.com/software/Arcgis]).

The results of the seven cluster *K*-means is presented in Fig. 5a. A description of the physical characteristics of each cluster was obtained based on interpretation of boxplots showing the correlation between the original 17 abiotic input variables and each of the seven clusters (Fig. 6). Four of the seven clusters (1, 4, 5, and 6) demonstrated a clear, distinct suite of physical conditions.

Clusters 1 and 6 were characterised by the prevalence of relatively strong bottom currents in a northerly (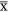  = 0.05 m/s) and south-easterly (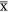 = 0.1 m/s) direction respectively. These currents appeared to be associated with large-scale, deep-sea topographic features (especially cluster 6). Cluster 4 was regionally widespread but locally spatially constrained. It demonstrated no dependence on depth or oceanography but was dominated by locally complex seabed topography. Cluster 4 exhibited steep slopes ( > 10°), high terrain ruggedness (rugosity) and a large range in curvature and topographic position values suggesting a region of topographic peaks and depressions. Cluster 5 was the most spatially discrete cluster and was characterised by shallow water; flat terrain; large annual temperature range; low salinity; and high primary productivity. We interpret this cluster as the continental shelf of South Georgia and Shag Rock.

The three remaining clusters (clusters 2, 3 and 7) are more difficult to constrain. All three clusters have a deep-sea element (depths > 2500 m). Cluster 3 had a large but northerly-restricted distribution. It exclusively represented deep-sea environments with low sea-bed temperature (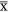 = 0.5 °C), but was primarily defined by its significantly higher sea-surface primary productivity (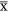 = 420 Mg C/m2/day). Cluster 2 also represented exclusively deep-sea (2500–4500 m) environments with low sea-bed temperature (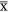 = 0.75 °C). It had a largely, but not exclusively southern spatial distribution. However it was the drivers of other clusters (primary productivity for cluster 3 and temperature for cluster 7) that distinguished them from it rather than its own characterisation that sets it apart. As such depending on the interpretation of the importance of surface primary productivity on deep-sea benthic environments (cluster 3) it is entirely plausible to group clusters 2 and 3 together as a generic deep-sea, flat abyssal plain with stable low temperature.

Cluster 7 was characterised by annually-stable, warmer sea-bed temperatures (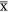 = 1.4 °C). Though mostly occurring in depths of 2000–3000 m the overall bathymetric range of the cluster was wide with the deeper extremities of the South Georgia shelf and the lower gradient regions of the slope represented by cluster 7. The only bathymetric exclusion from cluster 7 were shelf environments with depths < 250 m.

#### Confidence map

**Figure 7:**
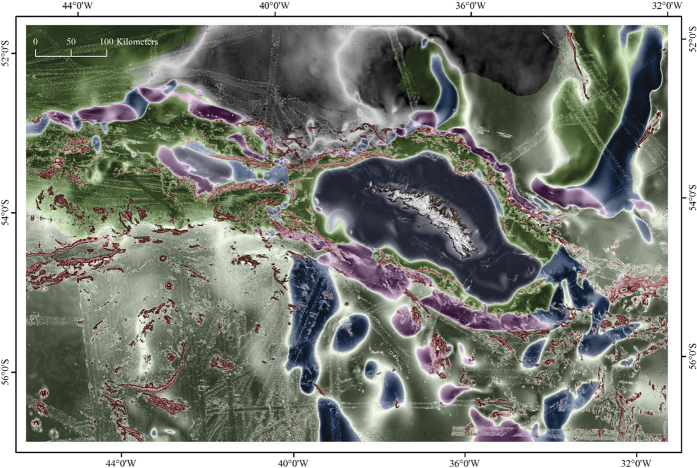
Confusion Index map quantifying clustering uncertainty across the study region. The confusion index is overlaid on the seven-cluster landscape map showing where zones of higher uncertainty (white) correspond with the boundaries between clusters and where there are instances of intra-cluster uncertainty. Data for figures gridded in R (version 3.0 [www.esri.com/software/Arcgis]) and visualised using ArcGIS (version 10.1 [www.esri.com/software/Arcgis]).

The confusion index map (Fig. 7) provided an assessment of the confidence values associated with the membership of each grid square to its given cluster defined through *K*-means partitioning. In general under a good cluster solution, high confusion values (denoted by white in Fig. 7) would be expected to only occur in the ‘transition zone’ on the boundaries between clusters. Confusion within clusters however would imply the clustering solution is not particularly stable and that those particular grid-cells do not have a strong affinity with a single *K*-means cluster.

Mapping the confusion indices revealed high confidence in the clustering of the South Georgia shelf (and to a lesser extent Shag Rocks). There was very little within-cluster confusion, which was restricted to the edges of cross-shelf troughs (presumably as a result of seabed topography), inshore shelf (presumably resulting from a distinct salinity and temperature gradients) and at a single anomaly on the south-western edge of the shelf break. With these exceptions the cluster is delineated by a well-defined uncertainty ‘halo’. Generally there was an increased level of background confusion (most notably in deep water) as a result of sampling artefact caused by high-resolution multibeam swath lines transecting regions of lower data quality. This was notable across clusters but was particularly apparent in the bottom-left quadrant of the plot that had been subject to a large-scale AWI multibeam survey (Fig. 2).

Both clusters defined by prevailing currents (clusters 1 and 6) were characterised by regions of well-defined zones of lower uncertainty. Cluster 3 was subdivided by a well-defined line of uncertainty forming an arc in the top-centre of the map. Inspection of the bathymetry dataset revealed the arc demarked the contour of a large topographic dome 100 km in diameter and 1000 m in height, with the uncertainty reflecting this change in topography (potentially conflicting with cluster 4). In contrast the boundary of clusters 2 and 3 in the top-right quadrant were not delineated by any uncertainty. The major driver of the separation of these two clusters was higher net primary productivity occurring in cluster 3. Inspection of the primary productivity data revealed an abrupt change in primary productivity at this boundary. This lack of a gradual change would explain the subsequent confidence in the cluster designation. The certainty of cluster 4 was hard to quantify given it consisted of predominantly spatially small topographic features. In general however the well constrained clusters of lower uncertainty delineated by narrow transition zones between clusters supports the *K*-means partition as robust physical landscape classification of the region.

#### Nested Hierarchical Clustering

Given the stability and spatially discrete nature of the shelf cluster, cluster 5 was selected to test the functionality of a nested hierarchical clustering solution. The original abiotic input datasets were clipped to the spatial extent of cluster 5. The landscape mapping protocol was then re-run to create a new set of shelf sub-clusters (Fig. 5b,c). Assessment of the shelf sub-clusters (Supplementary materials Fig. 2) revealed that annual temperature range, salinity, and slope dominated near-shore locations (cluster 2). A combination of depth (cluster 1), stronger current regimes (cluster 3 and 4), and complex topography (cluster 6) dominated on the outer shelf locations. A body of annually-stable cold bottom water drove designation of the large spatial coverage of sub-cluster 5. A third nested clustering of this sub-cluster 5 partitioned the shelf environment further still, delineating seven distinct clusters (Fig. 5c, Supplementary materials Fig. 3).

## Discussion

The TOPOGRID algorithm, as in previous studies^24^,^25^,^26^ proved a robust methodology for gridding a digital elevation model from a compilation of disparate datasets, integrating spatially discontinuous data with different sampling densities. The input bathymetry data varied considerably in terms of the state of its post-processing. Approximately ten iterative cycles were required to obtain a final DEM, cleaning most erroneous soundings. TOPOGRID was notably sensitive when interpolating joins between bathymetry datasets. Hence a large buffer of 500m was created to smooth the boundary zone.

When compiling and interpolating multiple disparate bathymetry datasets into a DEM, the effective resolution of each grid is limited to, but not necessarily equal to, the pre-defined resolution (in this case 100m). As such when the interpolated bathymetric grid is used to create derivative datasets for landscape mapping, areas where the interpolation is underpinned by many data points will contain far more detail (albeit limited to 100m resolution) than regions of sparse data which appear comparatively smooth. The effect of this is that these high-density data regions appear to have higher topographic complexity (e.g. high rugosity) when in fact the model is simply recording an artefact of sampling provenance. Rather than gridding the DEM at the resolution of the coarsest resolution data (in this case ~900 m), and in doing so removing fine-scale topographic details, we advocate the high-resolution analysis undertaken here with the caveats discussed taken into account when qualitatively assessing partitioning of environmental parameters.

The statistical approach to marine landscape mapping adopted in this study was proposed by Verfaillie *et al*.^18^. The utilisation of a statistical approach is intended to remove highly subjective decisions associated with classical landscape mapping protocol^42^,^43^, namely the selection of ecologically relevant abiotic input variables and the classifying of those variables into relevant classes. An objective means of defining partitions in the physical environment should be considered particularly useful for regions such as South Georgia where proposed and reviewed hierarchical definitions^40^ have not yet been established. This study represents the first application of this methodology over such a large regional scale (10^7^ km^2^). The results demonstrate that a statistical protocol is highly effective at reducing large numbers of sometimes collinear environmental input variables to a smaller number of relevant principal components. It is also effective at defining the optimal number of clusters and the spatial delineation of those clusters.

Adopting a ‘blind’ statistical approach to the data partitioning is problematic, however, as it does not remove all need for subjective supervision of the abiotic input variables and a careful evaluation of the process and its results is necessary. Firstly, PCA will remove collinearity in the data but if the model input includes for example many oceanographic variables and few topographic derived variables, then the output will be oceanographic-centric placing greater emphasis on the importance of these variables than anything else. Hence a balanced choice of input variables is needed. Secondly, *K*-means finds similar sized clusters based on spherical partitions in the multi-dimensional PC space. This is not always a correct representation of the reality, as meaningful clusters can vary in spatial extent and shape: for example, large numbers of data points might be clustered as deep-sea environments with fairly wide-ranging characteristics, whilst smaller rocky outcrops that punctuate that homogenous region would have much tighter environmental constraints. An alternative approach could be density-based spatial clustering (e.g. DBSCAN^44^), in which clusters are not restricted to a spherical designation. Such an approach may also be advantageous to large-scale datasets due to the ‘noise’ of data outliers that exists in datasets of this scale. The disadvantage of such an approach, however, is the requirement for a definition of high point-density. Finally, the protocol ensures that input variables are standardised and given equal weighing in the PCA. Not all input variables will drive biological distribution patterns to the same degree, but for areas that have not been studied much before, it is impossible to estimate these varying degrees of importance *a priori*.

To resolve these problems, our protocol is based on iterative refinement of each cluster solution. So whilst the process starts as an unsupervised classification based on statistical clustering, the resultant landscape map is qualitatively assessed on the basis of perceived biological, oceanographic and geomorphological meaningfulness. Any input variable deemed to be confounding the results is removed from the analysis and the protocol is re-run. For example, after the first iteration of this analysis, slope aspect was excluded from the analysis as deep-sea environments with relatively low slope gradients (<3°) were being partitioned based on minor variations in orientation of the slope. The aim of this approach is to strip down the analysis to retain only partitions with potential ecological relevance at a broad spatial scale. The process can then be re-run over iteratively smaller spatial subsets forming a nested hierarchical landscape classification (Fig. 5). As the importance of input variables as ecological drivers is scale-dependent, all input variables were included at the start of each new clustering level. This approach retains broad-scale gradients driving clusters across the entire region whilst identifying finer-scale geomorphic features nested within these broad-scale clusters.

A significant omission from this analysis is substrate type, which has been shown as a major driver of species composition^45^. It was not included in this analysis given the limited spatial extent of the sedimentology dataset in the region, and as such the difficulty in interpolating across such a wide area. Backscatter data were available for the regions with multibeam coverage (see Fig. 2), but interpretation was particularly sensitive to ship-specific calibrations which made a backscatter compilation problematic. It was our assessment, therefore, that the inclusion of geomorphology data layers in the PCA (slope angle, topography, rugosity) should effectively represent different sediment regimes in the analysis. For example homogenous flat regions are likely to be mud at South Georgia and steep escapements hard substrate. The downside of this approach is the spatial scale of the analysis will tend to pick out large-scale features (e.g. large escarpments, canyons, and gullies) but not sedimentary features with lower topographic profiles such as moraine fields, which have been suggested as important driver of marine biodiversity^46^.

Our results demonstrate that the iterative unsupervised landscape mapping protocol is effective at creating meaningful (Fig. 5) statistically stable (Fig. 7) partitions. Depth, current, and seabed topography are all shown to drive broad-scale clustering. At a finer scale on the South Georgia shelf, current regimes were shown to be less important with clustering driven by depth, sea-bed temperatures, coastal salinity, and topography (Fig. 5b,c, Supplementary materials 1 and 2). Taken in isolation this analysis provides valuable information pertaining to the nature of different physical habitats, their spatial distribution, and degree of heterogeneity^20^. In itself, this provides useful information for policy makers in terms of physical proxies for faunal richness with application in predictive modelling of taxa, functional traits, assemblages, and diversity^13^,^21^,^47^.

The next step will now be to quantify the biological meaningfulness of our physical landscape partitions. In the first instance, this will involve the inclusion of a regional biological dataset into the analysis^22^, to test for correlations between our proposed hierarchical clusters and biogeographical trends. The advantage of a landscape mapping approach for underpinning large-scale MPAs such as at South Georgia and South Sandwich Islands (SGSSI), as opposed to more holistic bottom-up approaches that integrate biological data in the analysis from the outset^48^,^49^,^50^, is largely on account of the spatial scale of the analysis. For example, although at South Georgia more than 25,000 biological point samples have been recorded^22^, spread over an area in excess of 1 million km^2^, the data exist at a spatial scale that is of limited application to draw statistically meaningful conclusions. Furthermore knowledge of benthic communities rapidly declines as a function of distance from the South Georgia continental shelf, with significant paucity in sampling of the region’s deep-sea environments. The biological data will be invaluable, however, in testing the output of the model to see how well it acts as a proxy for biological distributions. In doing this it will be important to better constrain other confounding influences on regional biogeography such as, paleo-environment and the presence of glacial refugia during the last glacial maximum^51^; the impact of iceberg scouring on biological communities^52^,^53^; and the effect of bathymetric divides (such as between South Georgia and Shag Rocks) as barriers to genetic transfer^54^,^55^.

Given sampling effort for many groups is uneven and most species in the region are rare, inferring distribution patterns at high taxonomic resolution (i.e. genus or species) over a large spatial extent will be problematic. More achievable (and perhaps with greater application in spatial planning) would be the mapping of a standardised (for sampling effort) measure of species level biodiversity to identify the presence of ‘biodiversity hotspots’ and correlate these with our physical landscape clusters. This approach would be helpful in understanding the role of habitat heterogeneity as a potential proxy for biodiversity (i.e. beta diversity). In addition to this, it would also be feasible to categorise certain taxa into functional groups (based on traits such as feeding, locomotive and reproductive strategies) and assess the relationship between these faunal aggregations and the clusters solutions. This could be used to assess the distributions of vulnerable marine ecosystems or habitat forming taxonomic groups.

It is also important to consider what the priorities are in terms of marine management. For example is it the presence of rare or endemic species ? If so then the methodology will have to reflect the fact that most species are rare^22^, so distribution is not well constrained. Is it the presence of species richness zones ? Or the presence of habitat forming fauna including vulnerable marine ecosystems (VMEs) such as coral gardens and sponge assemblage ? Alternatively benthic communities that offer an ecosystem service such as fisheries or carbon sequestration^46^ may be considered of importance to identify. The marine landscape map produced here provides a first-level, baseline picture of the spatial pattern in the marine regions around South Georgia, and can be used as a tool to start developing a method to answer each of the questions above.

## Conclusions

The methodology presented here provides an objective assessment of the physical attributes of the benthic environment over nested spatial scales, providing analysis from broad-scale drivers of biogeography such as large-scale (10–100 s km) abiotic gradients (e.g. depth and temperature) to smaller-scale features (100 m–1 kms) such as local topography. Currently this level of analysis on the factors driving biogeography is lacking from most marine spatial planning frameworks. We argue that it provides the potential for a holistic overview of the marine environment, and meaningful information to aid policy-makers to manage the region’s marine environment. Furthermore the approach is adaptable to different input variables and as such transferable globally to different proposed or established MPAs.

## Additional Information

**How to cite this article**: Hogg, O. T. *et al*. Landscape mapping at sub-Antarctic South Georgia provides a protocol for underpinning large-scale marine protected areas. *Sci. Rep*. **6**, 33163; doi: 10.1038/srep33163 (2016).

## Supplementary Material

Supplementary Information

## Figures and Tables

**Table 1:** Abiotic variables included in the landscape mapping analysis.

Abiotic Variables	Description	Unit	Scale
Topography			
Digital elevation model (DEM) of bathymetry	Bathymetric compilation of multibeam, singlebeam and soundings data (see Table 1 for sources) interpolated using TOPOGRID algorithm.	m	100 m
Slope	A first derivative of DEM representing the rate of change in depth from one cell to its neighbours.	°	100 m
Terrain Ruggedness Index	A measure of rugosity calculated as the ratio of the three-dimensional DEM surface area to the two-dimensional planar area of a cell.	**—**	100 m
Eastness = sin(aspect/57.296)	A first derivative of DEM providing a measure of the easterly orientation of the slope on a continuous scale (−1 to + 1).	**—**	100 m
Northness = cos(aspect/57.296)	A first derivative of DEM providing a measure of the northerly orientation of the slope on a continuous scale (−1 to + 1).	**—**	100 m
Profile curvature	A second derivative of DEM measuring the rate of change in the slope gradient.	**—**	100 m
Topographic Position Index (TPI)	A measure of whether a cell is positioned on a topographic peak, in a depression on in a region of constant gradient.	**—**	100 m
Satellite derived variables			
Net primary productivity	5-year mean net primary productivity calculated using VGPM, a global “chlorophyll-based” model that estimate net primary production from chlorophyll using a temperature-dependent description of chlorophyll-specific photosynthetic efficiency^31^.	Mg C/m^2^/day	1/12°
Oceanography	Three year means derived from the Proudman Oceanographic Laboratory Coastal Ocean Modelling System (POLCOMS) South Georgia high-resolution dataset^29^.		
Summer Seabed Temperature	Three-year austral summer (Dec–Feb) bottom temperature mean.	°C	2800 m
Summer Seabed Salinity	Three-year austral summer (Dec–Feb) bottom salinity mean.	PSU	2800 m
Summer Seabed Current U	Three-year austral summer (Dec–Feb) mean measure of the easterly orientation of the current on a continuous scale (−1 to + 1).	**—**	2800 m
Summer Seabed Current V	Three-year austral summer (Dec–Feb) mean measure of the northerly orientation of the current on a continuous scale (−1 to + 1).	**—**	2800 m
Summer Seabed Current Magnitude	Three-year austral summer (Dec–Feb) mean measure of current magnitude	m/s	2800 m
Winter Seabed Temperature	Three-year austral winter (Jun–Aug) bottom temperature mean.	°C	2800 m
Winter Seabed Salinity	Three-year austral winter (Jun-Aug) bottom salinity mean.	PSU	2800 m
Winter Seabed Current U	Three-year austral winter (Jun–Aug) mean measure of the easterly orientation of the current on a continuous scale (−1 to + 1).	**—**	2800 m
Winter Seabed Current V	Three-year austral winter (Jun–Aug) mean measure of the northerly orientation of the current on a continuous scale (−1 to + 1).	**—**	2800 m
Winter Seabed Current Magnitude	Three-year austral winter (Jun–Aug) mean measure of current magnitude	m/s	2800 m
Seabed Temperature Range	Temperature differential between three-year summer and winter means.	°C	2800 m

**Table 2:** Component matrix showing correlation between the Varimax rotated PCs and the original input variables.

Abiotic Variables	PC1	PC2	PC3	PC4	PC5	PC6
Depth	**−0.652**	**—**	**—**	**—**	**0.673**	**—**
Slope	**—**	**—**	**—**	**0.958**	**—**	**—**
Terrain Ruggedness Index	**—**	**—**	**—**	**0.959**	**—**	**—**
Curvature	**—**	**—**	**—**	**—**	**—**	**0.807**
Topographic Position Index	**—**	**—**	**—**	**—**	**—**	**0.812**
Primary Productivity	**—**	**—**	**—**	**—**	**—**	**—**
Summer Seabed Temperature	**—**	**—**	**—**	**—**	**0.926**	**—**
Summer Seabed Salinity	**0.951**	**—**	**—**	**—**	**—**	**—**
Summer Seabed Current U	**—**	**0.875**	**—**	**—**	**—**	**—**
Summer Seabed Current V	**—**	**—**	**0.968**	**—**	**—**	**—**
Summer Seabed Current Magnitude	**—**	**0.861**	**—**	**—**	**—**	**—**
Winter Seabed Temperature	**—**	**—**	**—**	**—**	**0.970**	**—**
Winter Seabed Salinity	**0.933**	**—**	**—**	**—**	**—**	**—**
Winter Seabed Current U	**—**	**0.882**	**—**	**—**	**—**	**—**
Winter Seabed Current V	**—**	**—**	**0.974**	**—**	**—**	**—**
Winter Seabed Current Magnitude	**—**	**0.861**	**—**	**—**	**—**	**—**
Seabed Temperature Range	**−0.763**	**—**	**—**	**—**	**—**	**—**
Variance Explained (%)	16.60%	18.40%	11.70%	11.80%	14.80%	7.70%
Cumulative Variance (%)	16.60%	35.00%	46.70%	58.50%	73.30%	81.00%
Eigenvalues	**3.7053**	**3.3623**	**2.0678**	**1.8257**	**1.5012**	**1.3138**

High factor loads (r < −0.6 or r > 0.6) are highlighted in bold; Low factor loads (r < −0.3 or r > 0.3) are omitted.
