# Supplementary material for: Landscape mapping at sub-Antarctic South Georgia provides a protocol for underpinning large-scale marine protected areas
Source: Sci Rep. 2016 Oct 3;6:33163. doi: 10.1038/srep33163 (PMC5046182; doi:10.1038/srep33163)
Supplement: Supplementary Information [file srep33163-s1.pdf]

Landscape mapping at sub-Antarctic South Georgia provides a protocol for underpinning large-scale marine protected areas (supplementary materials)

OLIVER T. HOGG[1, 2, 3, *], VEERLE A.I. HUVENNE[2], HUW J. GRIFFITHS[1], BORIS DORSCHEL[4] and KATRIN LINSE[1]

[1] British Antarctic Survey, Natural Environment Research Council, High Cross, Madingley Road, Cambridge, CB3 OET, U.K.

[2] National Oceanography Centre, University of Southampton Waterfront Campus, European Way, Southampton SO14 3ZH, Southampton, U.K.

[3] University of Southampton, Waterfront Campus, European Way, Southampton SO14 3ZH, U.K.

[4] Alfred Wegener Institute, Helmholtz Centre for Polar and Marine Research, Mitte, Am Alten Hafen 26, 27568 Bremerhaven, Germany.

[*] Corresponding author: olgg@bas.ac.uk

Survey ID	Data Type	Year	Source
**BAS DATA**			
**JR287**	Kongsberg EM122 multibeam	2013	BAS
**JR257-272A-254E**	Kongsberg EM122 multibeam	2012	BAS
**JR262-260A**	Kongsberg EM122 multibeam	2011	BAS
**JR184**	Kongsberg EK60 echo sounder	2007	BAS
**JR168-167**	Kongsberg EM120 multibeam	2007	BAS
**JR167-168**	Kongsberg EM120 multibeam	2007	BAS
**JR161**	Kongsberg EM120 multibeam	2006	BAS
**JR152-159**	Kongsberg EM120 multibeam	2006	BAS
**JR149**	Kongsberg EM120 multibeam	2006	BAS
**JR151**	Kongsberg EM120 multibeam	2006	BAS
**JR134**	Kongsberg EM120 multibeam	2005	BAS
**JR114-121**	Kongsberg EM120 multibeam	2005	BAS
**JR134**	Kongsberg EM120 multibeam	2005	BAS
**JR130**	Kongsberg EM120 multibeam	2005	BAS
**JR77-78**	Kongsberg EM120 multibeam	2004	BAS
**JR107**	Kongsberg EM120 multibeam	2004	BAS
**JR109**	Kongsberg EM120 multibeam	2004	BAS
**JR116**	Kongsberg EM120 multibeam	2004	BAS
**JR82**	Kongsberg EK60 echo sounder	2004	BAS
**JR92**	Kongsberg EK60 echo sounder	2003	BAS
**JR93**	Kongsberg EM120 multibeam	2003	BAS
**JR100**	Kongsberg EK60 echo sounder	2003	BAS
**JR103**	Kongsberg EM120 multibeam	2003	BAS
**JR72**	Kongsberg EM120 multibeam/EK500	2003	BAS
**JR60**	Kongsberg EK60 echo sounder	2002	BAS
**JR69**	Kongsberg EM120 multibeam	2001	BAS
**AWI DATA**			
**ANT29_4**	Hydrosweep DS3	2013	AWI
**ANT29_5**	Hydrosweep DS3	2013	AWI
**ANT27_3**	Hydrosweep DS3	2011	AWI
**ANT23_5**	Hydrosweep DS2	2006	AWI
**ANT23_7**	Hydrosweep DS2	2006	AWI
**ANT22_4**	Hydrosweep DS2	2005	AWI
**ANT22_2**	Hydrosweep DS2	2004	AWI
**ANT19_4**	Hydrosweep DS2	2002	AWI
**ANT19_5**	Hydrosweep DS2	2002	AWI
**ANT18_4**	Hydrosweep DS2	2001	AWI
**ANT15_4**	Hydrosweep DS2	1998	AWI
**ANT14_3**	Hydrosweep DS1	1997	AWI
**ANT15_2**	Hydrosweep DS2	1997	AWI
**ANT12_3**	Hydrosweep DS1	1995	AWI
**ANT11_3**	Hydrosweep DS1	1994	AWI
**ANT10_2**	Hydrosweep DS1	1992	AWI
**ANT10_5**	Hydrosweep DS1	1992	AWI
**ANT08_3**	Hydrosweep DS1	1989	AWI
**ANT08_5**	Hydrosweep DS1	1989	AWI

**ANT06_3**	L3 ELAC Nautik SeaBeam	1987	AWI
**ANT04_4**	L3 ELAC Nautik SeaBeam	1986	AWI
**ANT04_3**	L3 ELAC Nautik SeaBeam	1985	AWI
**OTHER CRUISES**			
**NBP 1408**	Kongsberg EM122 multibeam	2014	UoT
**BAS/UK Hydrographic Office tracks**	Kongsberg EA600 single-beam	-	UKHO
**HO chart no. 3596**	Soundings from scanned charts	-	UKHO
**HO chart no. 3597**	Soundings from scanned charts	-	UKHO
**Fisheries data**	Single-beam echo sounders (FV Argos, Helena, FPV, Dorada)	2003-04/2015	GSGSSI
**HMOI_1285**	-	2009	BAS/UKHO
**HMOI_1128**	-	2006	BAS/UKHO
**HMOI_1129**	-	2006	BAS/UKHO
**HMOI_1130**	-	2006	BAS/UKHO
**PRTR_Douglas_Strait**	-	2014	HMS Protector
**AMLR95**	SeaBeam	1994	Surveyor
**NBP9705**	SeaBeam 2112	1997	LDEO MGDS
**NBP0506**	Kongsberg EM120 multibeam	2005	LDEO MGDS
**NBP0603**	Kongsberg EM120 multibeam	2006	LDEO MGDS
**NBP0805**	Kongsberg EM120 multibeam	2008	LDEO MGDS
**Hes97**	-	1996	IACT
**GEBCO database**	Global bathymetric compilation	2014	GEBCO_2014

Supplementary Materials Table 1: Data sets used in the South Georgia bathymetric compilation. BAS is the British Antarctic Survey; AWI is the Alfred Wegener Institute; UoT is the University of Texas; UKHO is the UK Hydrographic Office; GSGSSI is the Government of South Georgia & South Sandwich Islands; LDEO MGDS is the Lamont-Doherty Earth Observatory Marine Geoscience Data System; IACT is the Instituto Andaluz de Ciencias de la Tierra; GEBCO is The General Bathymetric Chart of the Oceans with data derived from The GEBCO_2014 Grid, version 20141103 (http://www.gebco.net).

Supplementary Materials Figure 1: Depth- profiles of new bathymetric compilation showing (A) Complex topography of the South Georgia shelf punctuated by cross shelf troughs, (B) Abrupt change in gradient of the southern South Georgia shelf break, (C) More gentle transition from shelf to slope at the northern South Georgia shelf-break, (D) Transect south of the island showing topographic complexity in the deep-sea including sea mount, (E) Detail of deep-sea topographic feature, (F) Transect from Shag Rock (left) to South Georgia (right) showing deep ‘Western Gully’ divide between the two shelves.

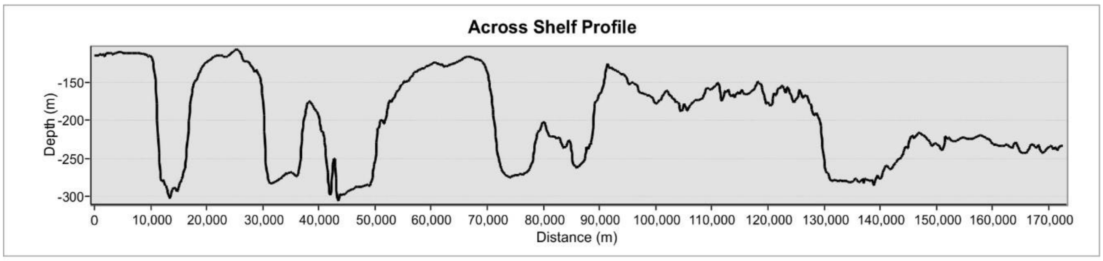
Across Shelf Profile
Depth (m)
-150
-200
-250
-300
0
10,000
20,000
30,000
40,000
50,000
60,000
70,000
80,000
90,000
100,000
110,000
120,000
130,000
140,000
150,000
160,000
170,000
Distance (m)

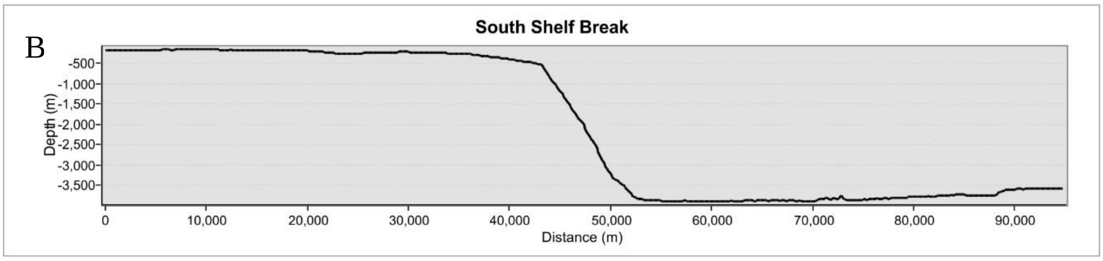
B
South Shelf Break
Depth (m)
-500
-1,000
-1,500
-2,000
-2,500
-3,000
-3,500
0
10,000
20,000
30,000
40,000
50,000
60,000
70,000
80,000
90,000
Distance (m)

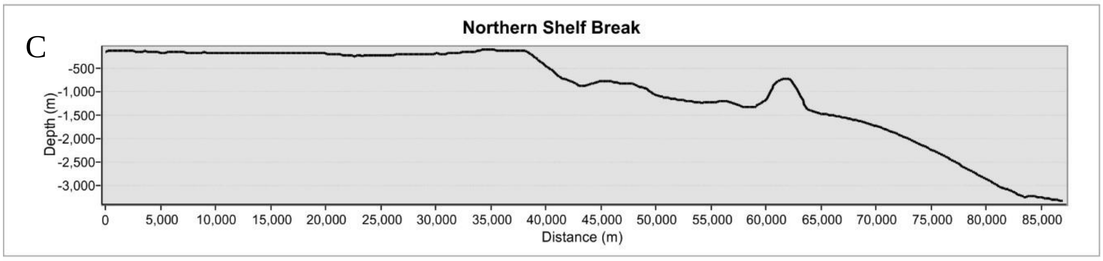
C
Northern Shelf Break
Depth (m)
-500
-1,000
-1,500
-2,000
-2,500
-3,000
0
5,000
10,000
15,000
20,000
25,000
30,000
35,000
40,000
45,000
50,000
55,000
60,000
65,000
70,000
75,000
80,000
85,000
Distance (m)

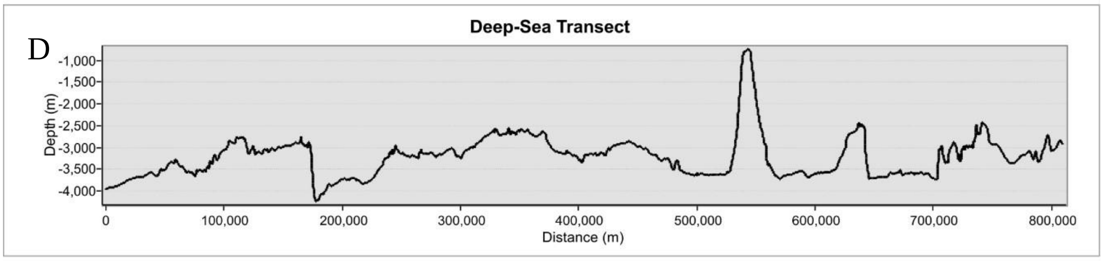
D
Deep-Sea Transect
Depth (m)
-1,000
-1,500
-2,000
-2,500
-3,000
-3,500
-4,000
0
100,000
200,000
300,000
400,000
500,000
600,000
700,000
800,000
Distance (m)

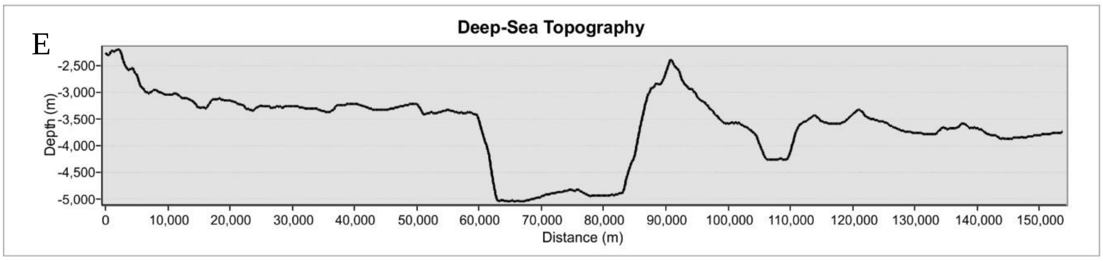
E
Deep-Sea Topography
Depth (m)
-2,500
-3,000
-3,500
-4,000
-4,500
-5,000
0
10,000
20,000
30,000
40,000
50,000
60,000
70,000
80,000
90,000
100,000
110,000
120,000
130,000
140,000
150,000
Distance (m)

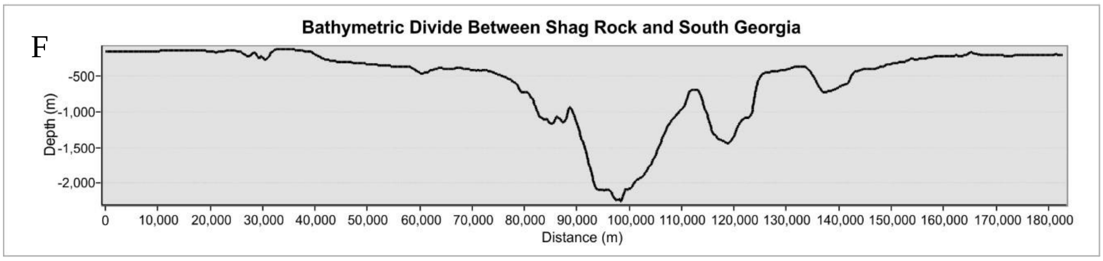
F
Bathymetric Divide Between Shag Rock and South Georgia
Depth (m)
-500
-1,000
-1,500
-2,000
0
10,000
20,000
30,000
40,000
50,000
60,000
70,000
80,000
90,000
100,000
110,000
120,000
130,000
140,000
150,000
160,000
170,000
180,000
Distance (m)

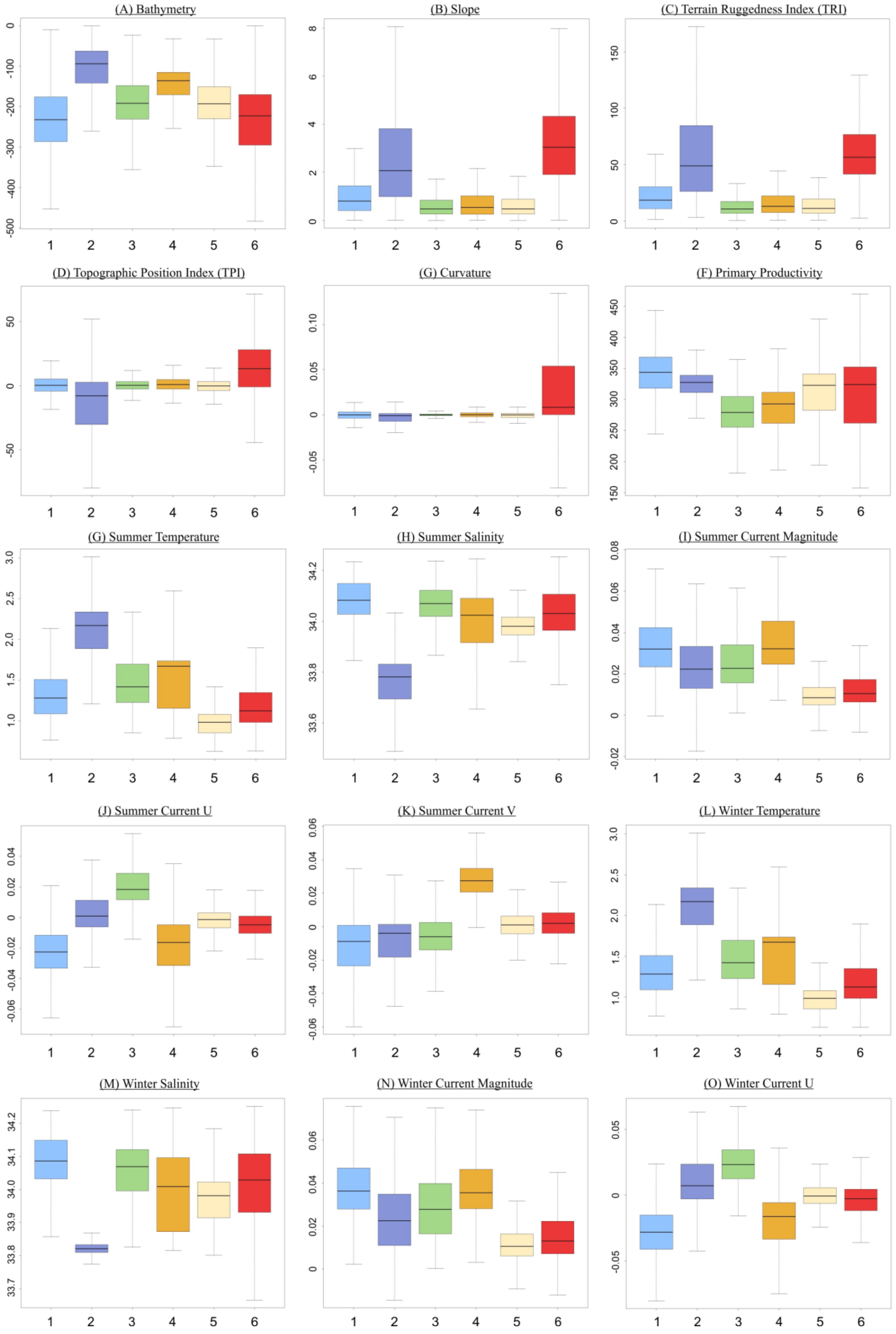
(A) Bathymetry
(B) Slope
(C) Terrain Ruggedness Index (TRI)
(D) Topographic Position Index (TPI)
(G) Curvature
(F) Primary Productivity
(G) Summer Temperature
(H) Summer Salinity
(I) Summer Current Magnitude
(J) Summer Current U
(K) Summer Current V
(L) Winter Temperature
(M) Winter Salinity
(N) Winter Current Magnitude
(O) Winter Current U
1 2 3 4 5 6

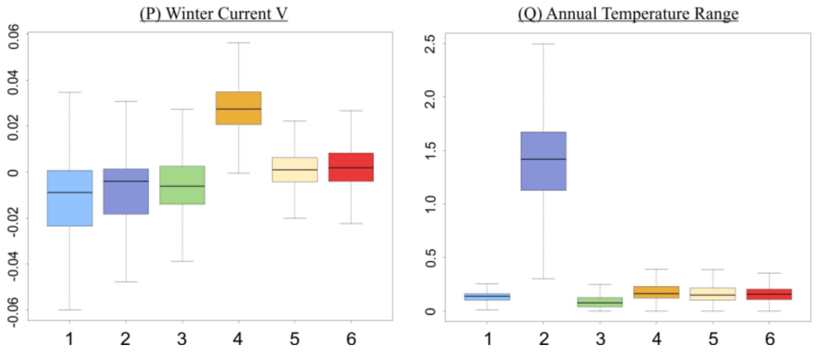

Supplementary Materials Figure 2: Box plots of k-means derived clusters from sub-cluster 5 (figure 5b) (X-axis) against the 17 original abiotic variables (excluding slope aspect) (Y-axis). Descriptions of each variable including their units are summarised in table 1. In each box plot the middle line represents the median, the upper and lower extent of the box represent the first and third quartiles. The whiskers are the maximum and minimum observed values (excluding statistical outliers - values >1.5 x the interquartile range). Box plot colours denote the corresponding landscape map cluster colours from figure 5b.

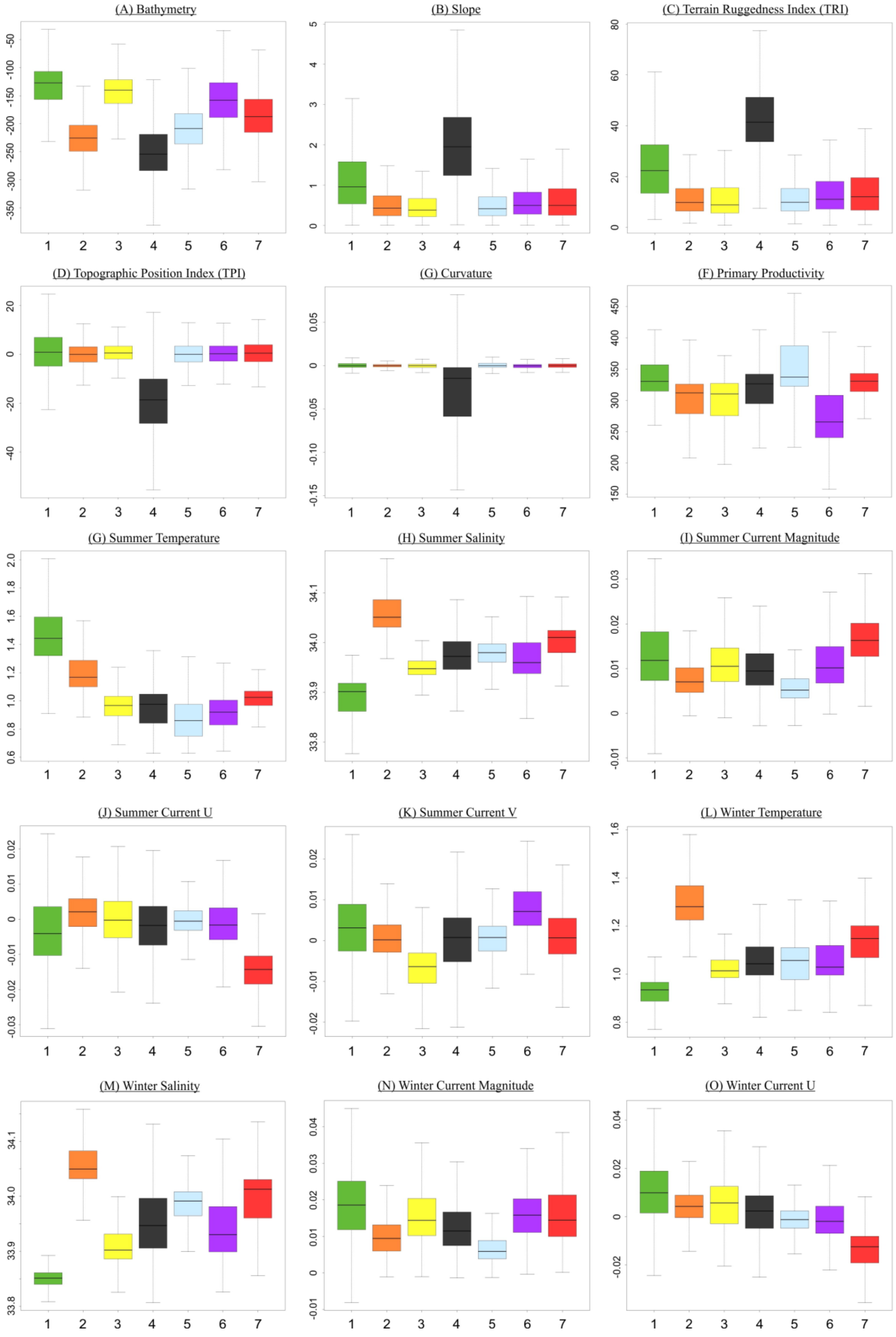
(A) Bathymetry
(B) Slope
(C) Terrain Ruggedness Index (TRI)
(D) Topographic Position Index (TPI)
(G) Curvature
(F) Primary Productivity
(G) Summer Temperature
(H) Summer Salinity
(I) Summer Current Magnitude
(J) Summer Current U
(K) Summer Current V
(L) Winter Temperature
(M) Winter Salinity
(N) Winter Current Magnitude
(O) Winter Current U

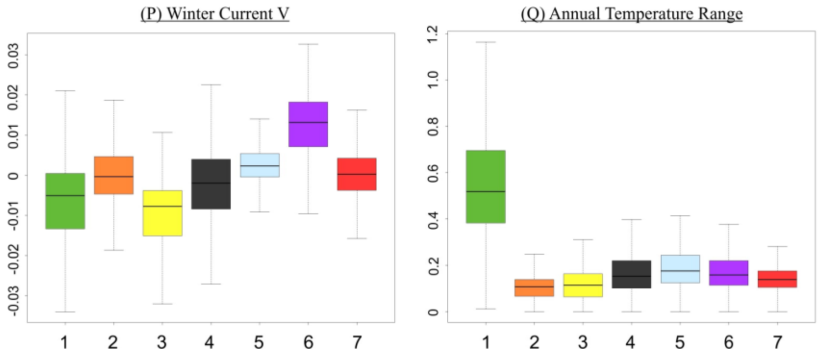

Supplementary Materials Figure 3: Box plots of k-means derived clusters from re-clustering of cluster 5 of sub-cluster 5 (figure 5c) (X-axis) against the 17 original abiotic variables (excluding slope aspect) (Y-axis). Descriptions of each variable including their units are summarised in table 1. In each box plot the middle line represents the median, the upper and lower extent of the box represent the first and third quartiles. The whiskers are the maximum and minimum observed values (excluding statistical outliers - values >1.5 x the interquartile range). Box plot colours denote the corresponding landscape map cluster colours from figure 5c.
